# Supplementary material for: Interaction and functional specialization across a distributed neural circuit for flexible task control in macaques
Source: Nat Commun. 2026 Jan 24;17:1063. doi: 10.1038/s41467-025-65766-0 (PMC12852943; doi:10.1038/s41467-025-65766-0)
Supplement: Supplementary file 1 — Supplementary Information [file 41467_2025_65766_MOESM1_ESM.pdf]

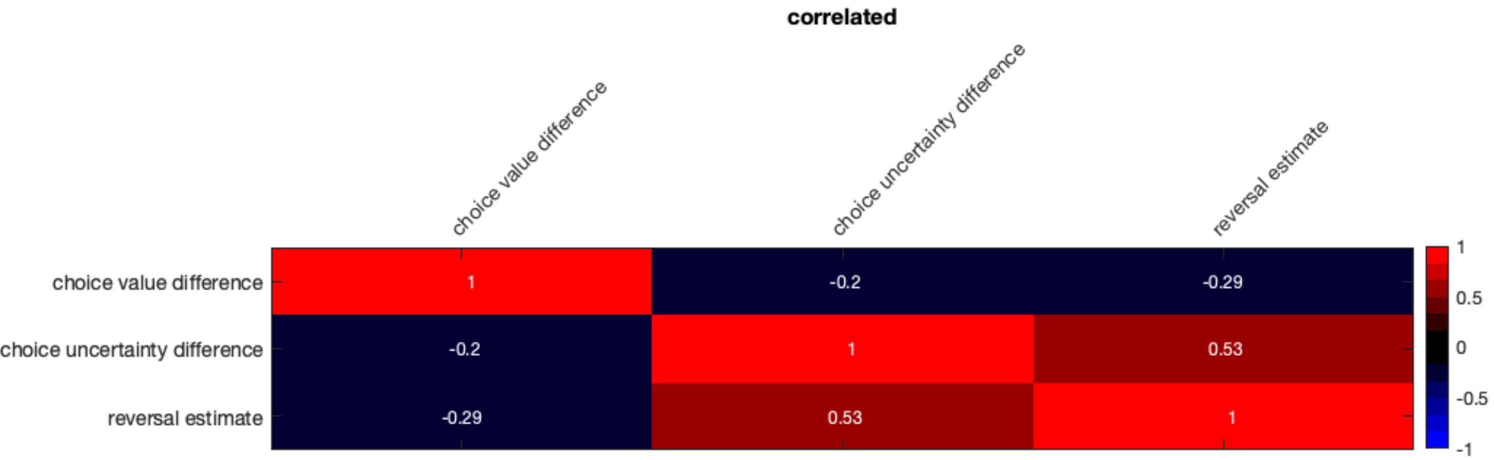
correlated
choice value difference
choice uncertainty difference
reversal estimate
1
-0.2
-0.29
-0.2
1
0.53
-0.29
0.53
1
1
0.5
0
-0.5
-1

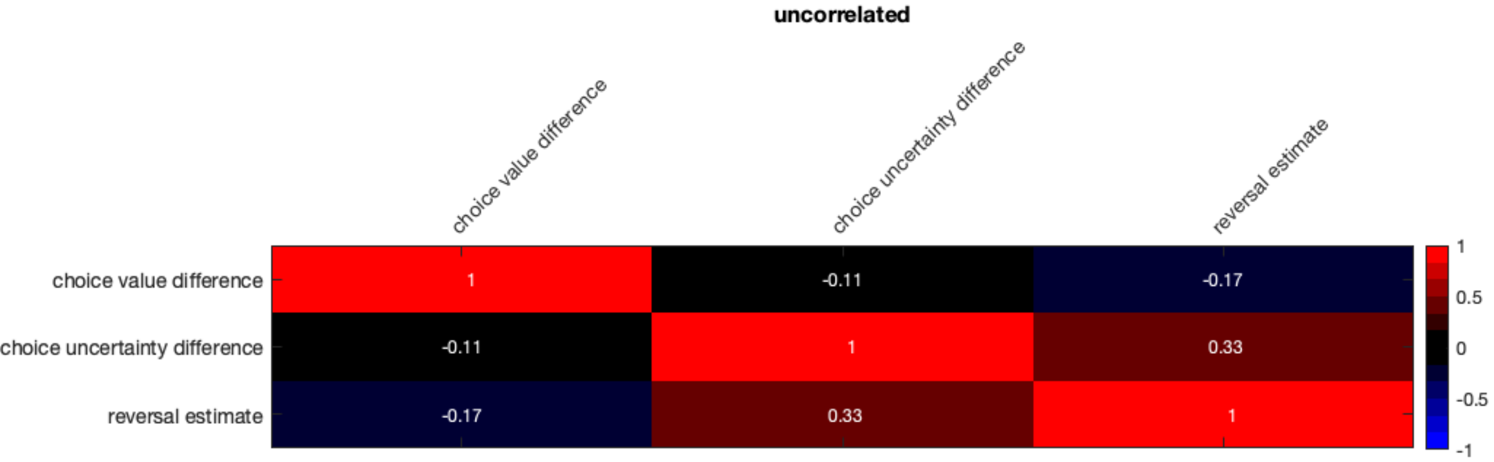
uncorrelated
choice value difference
choice uncertainty difference
reversal estimate
1
-0.11
-0.17
-0.11
1
0.33
-0.17
0.33
1
1
0.5
0
-0.5
-1

S1. Correlations between model parameters. Correlations between the differences in the values of the choices (the chosen option's probability of reward - the unchosen option's probability of reward), the differences in the uncertainties of these estimates (chosen uncertainty - unchosen uncertainty), and reversal estimate in the correlated and uncorrelated sessions.

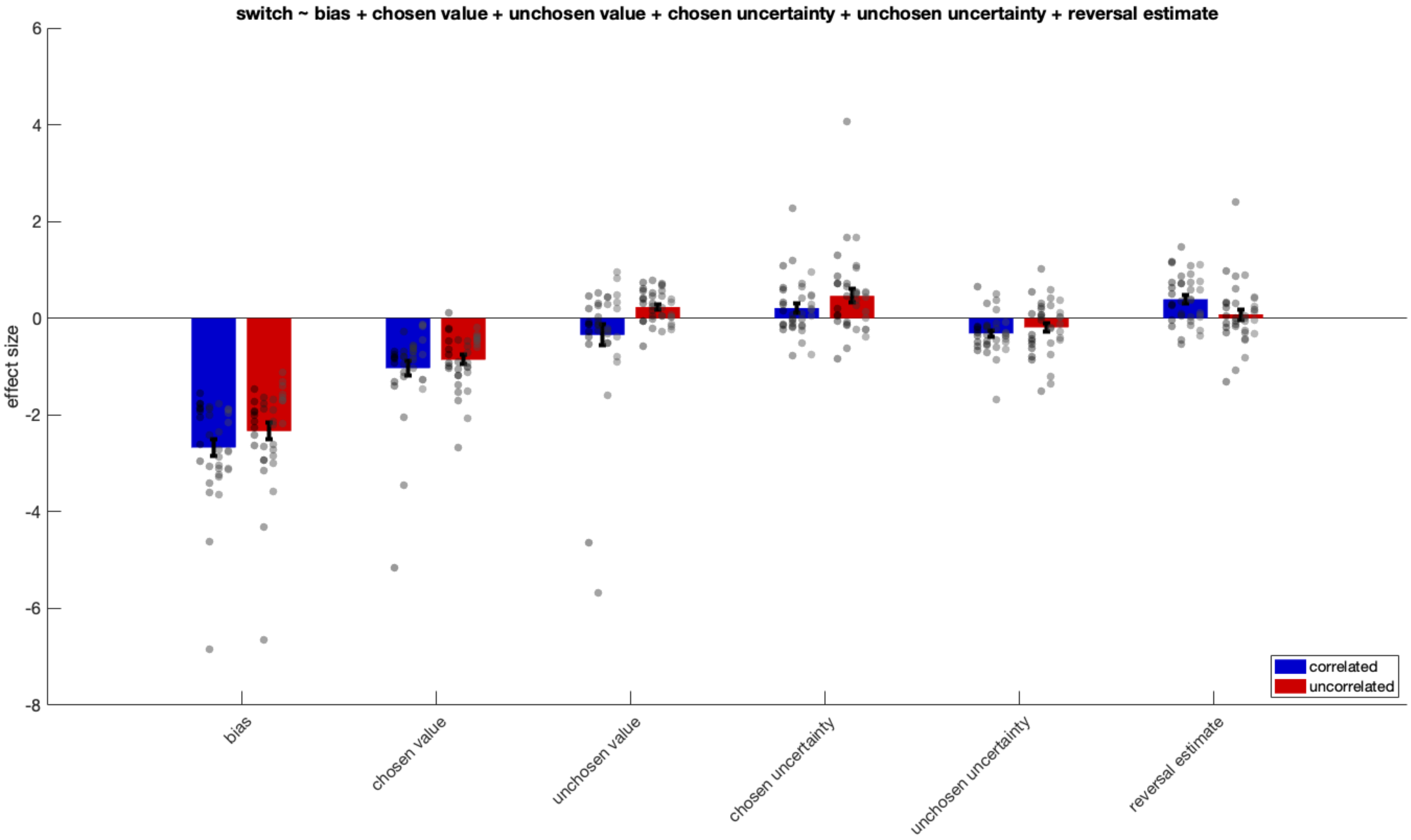

switch ~ bias + chosen value + unchosen value + chosen uncertainty + unchosen uncertainty + reversal estimate
effect size
6
4
2
0
-2
-4
-6
-8
bias
chosen value
unchosen value
chosen uncertainty
unchosen uncertainty
reversal estimate
correlated
uncorrelated

S2. An alternative analysis approach also suggests that reversal estimate has a greater influence on switching in correlated sessions. (A) Main figure 3 illustrates the influence on switching of the difference in the options' reward probabilities and the difference in the options' uncertainties. Here, by contrast, we show the separate influences on switching of the estimates of reward probability associated with each option, the chosen and the unchosen option, and the uncertainties on each of these estimates. Logistic regression analyses were applied to each session (illustrated by grey dots) from each animal (illustrated in three columns corresponding to each of the three animals) to illustrate the impact of the factors in terms of regression coefficients for each animal and session. Statistical inference was performed in the hierarchical LMEM2. Across both correlated and uncorrelated sessions, the higher the chosen option's reward probability, the lower the probability of switching (chosen value: χ^2_1=106.8956; p<0.001). There was no significant difference in the influence of this factor in the two session types (χ^2_1=2.2701; p=0.132). The unchosen option's reward probability was not, in general, across both correlated and uncorrelated sessions, associated with a consistent influence on switching. In uncorrelated sessions, monkeys were more likely to switch when the estimate of the alternative option's reward probability was higher while the opposite was the case in the correlated sessions; the difference between the influence of the unchosen option's reward probability was significantly greater in the uncorrelated sessions than in the correlated sessions (unchosen value: χ^2_1=13.6142; p<0.001). The influence of the uncertainty of the reward probability estimate for the chosen option was not consistent across correlated and uncorrelated sessions and so its effect, in general, was not significant (chosen uncertainty: χ^2_1=3.0703; p=0.078) but the effect was significantly greater in the uncorrelated sessions (χ^2_1= 7.2583; p<0.007). The influence of the uncertainty of the reward probability estimate for the unchosen option was consistent across both correlated and uncorrelated sessions and so its effect, in general, was significant (unchosen uncertainty: χ^2_1=14.4178; p<0.001) but the effect did not differ between correlated and uncorrelated sessions (χ^2_1=0.4069; p=0.5235). Reversal estimate continued to exert a significant influence on switching (χ^2_1=25.4413; p<0.001) which was greater in the correlated sessions (χ^2_1=28.8443; p<0.001).

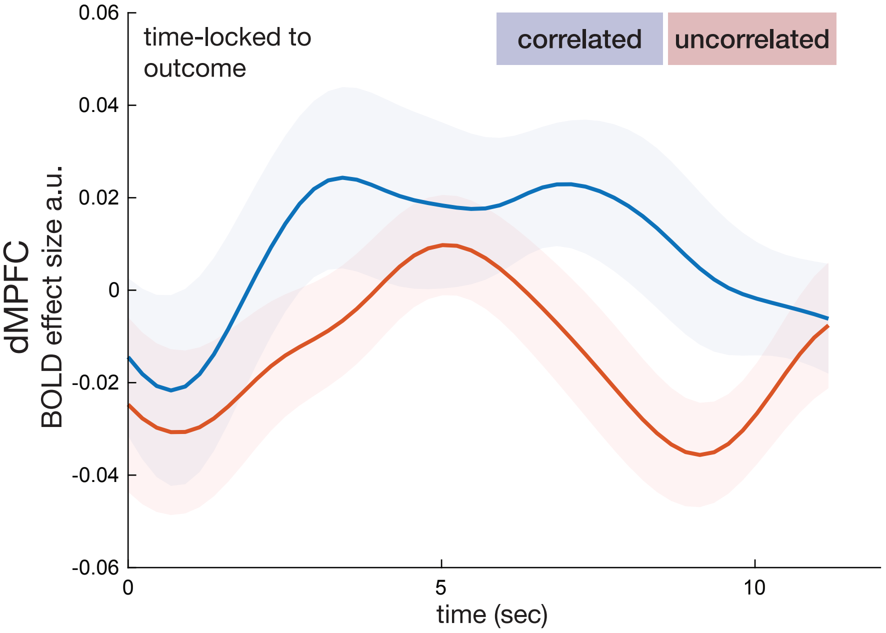
0.06
0.04
0.02
0
-0.02
-0.04
-0.06
time-locked to outcome
correlated
uncorrelated
dMPFC
BOLD effect size a.u.
0
5
10
time (sec)

S3 **Reversal DKL-related activity in dmPFC.** Reversal D_{KL}-related activity in anterior dmPFC (same location as figure 4B) began to appear shortly after feedback was obtained more prominently in correlated than uncorrelated sessions.

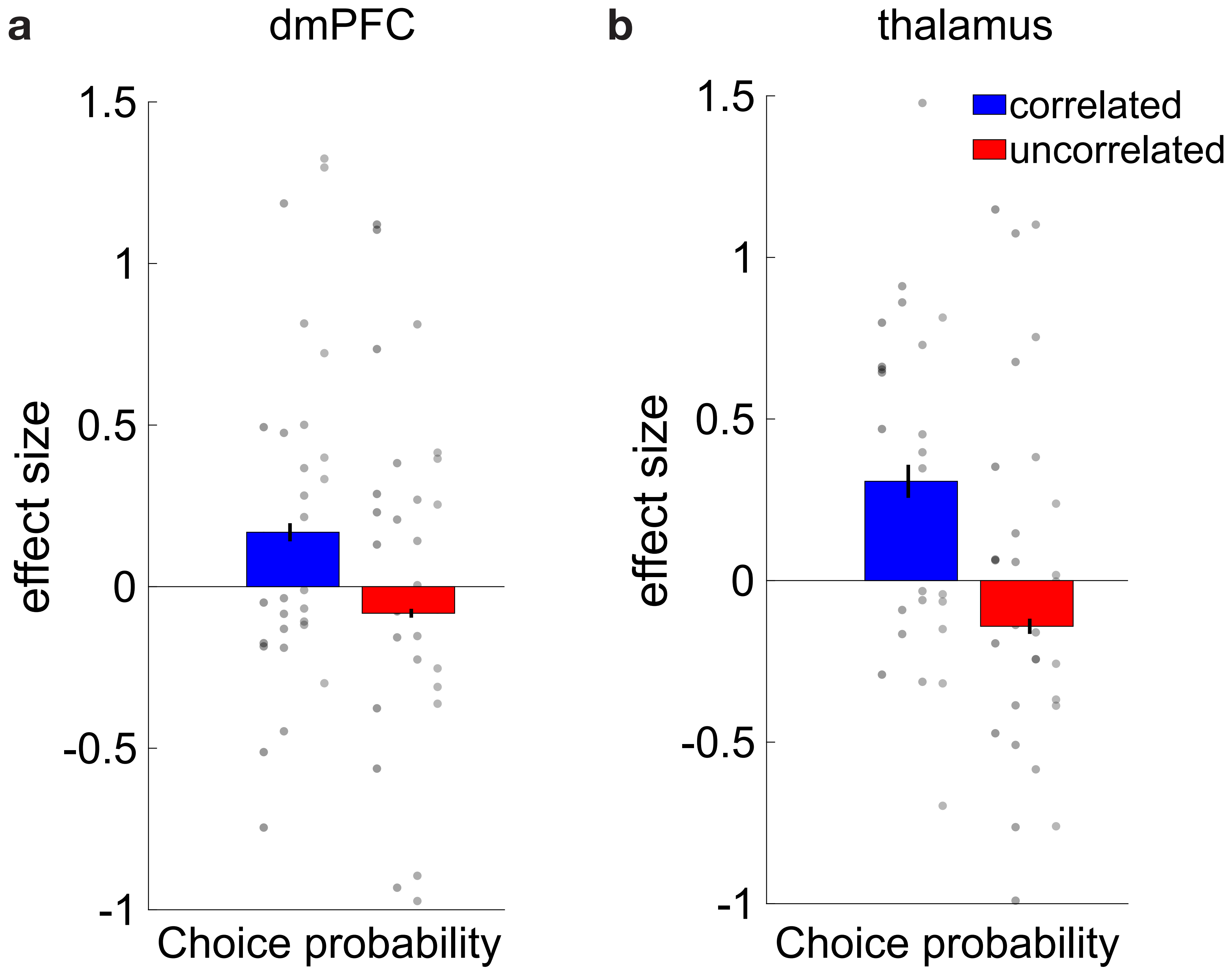

a
dmPFC
b
thalamus
correlated
uncorrelated
effect size
Choice probability
1.5
1
0.5
0
-0.5
-1

S4. Decision-related activity estimated under the assumption that monkeys make inferences about the value of unchosen options from their experience with chosen options. Given that animals infer a reversal in latent state, there are two ways that they might then establish a value for the unchosen option. They might try out the unchosen response and learn by observation what its value now is. Activity related to the chosen and unchosen values under this assumption is illustrated in figure 6. Alternatively, animals might make a second type of inference; in addition to inferring the change in latent state, they might infer a precise value about the value of the unchosen option on the basis that the values of the two choices were negatively correlated with one another; if they observed that the chosen value decreased, they might infer that the value of the unchosen option increased. Here, we therefore used a second version of Model 2 that incorporated the assumption that this second type of inference about the unchosen option might be made. In other words, If the animals learn from experience that one option that they have chosen recently has a probability, p, of being rewarded then, because the options' reward probabilities are negatively correlated, they should infer that the unchosen option's probability of reward is 1-p. Decision-related activity in dmPFC (A) and anterior and medial thalamus (B) is estimated under the assumption that monkeys make such inferences in correlated sessions. Because the regressors for the two options are now perfectly negatively correlated with one another it is not possible to look at neural activity that is specifically related to one option or the other (because activity that appears positively related to the value of one option is equally negatively related to the value of the other option) but only to look at activity related to the difference in the options' reward probabilities (chosen option's reward probability – unchosen option's reward probability) and so this is what is illustrated here. The blue bars in this figure should, therefore, approximately correspond to the difference between the values of each pair of blue bars in Figures 6C-D where we can look separately at the value of the chosen option and the value of the unchosen option. This is, indeed, the case.

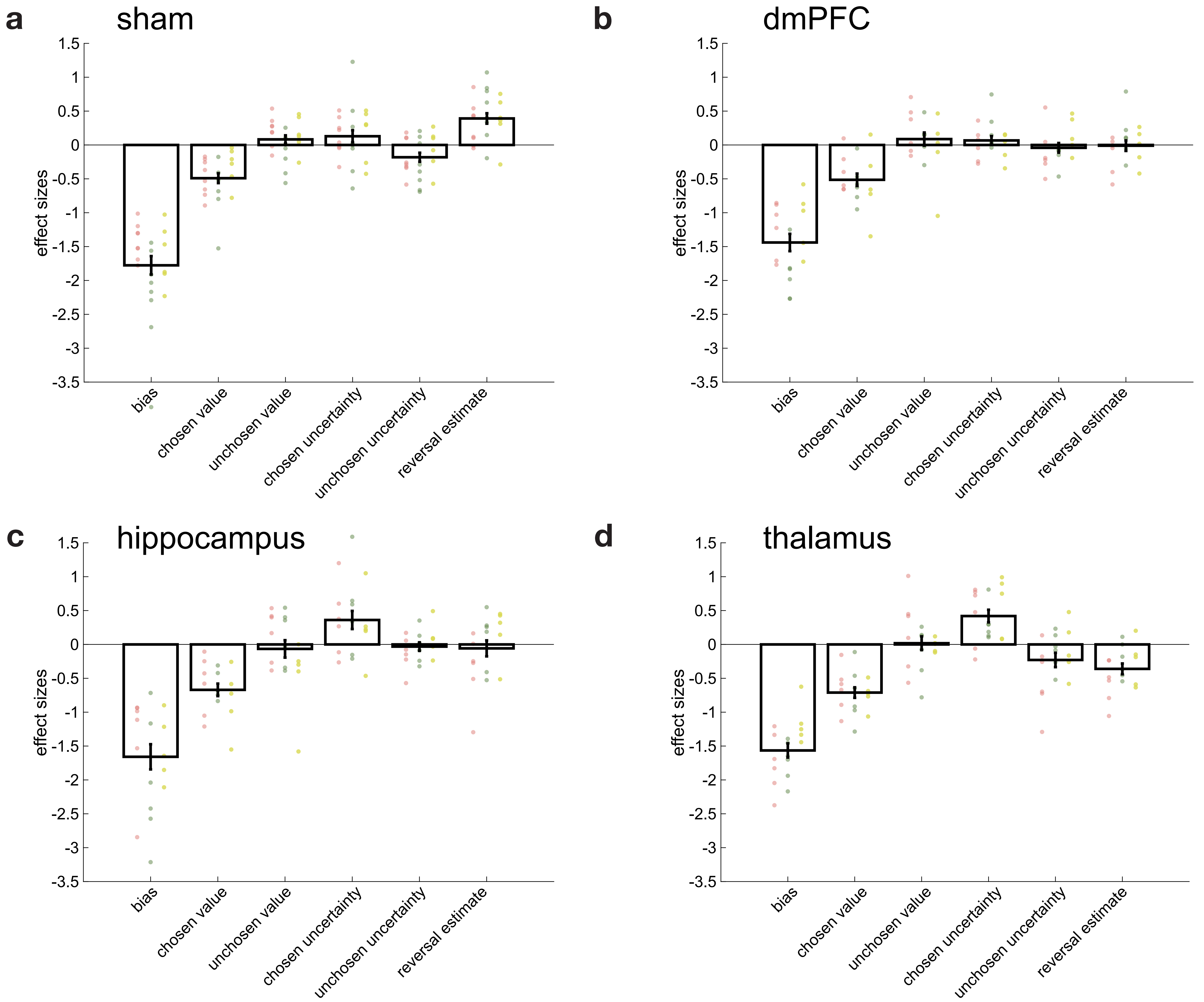
a
sham
b
dmPFC
c
hippocampus
d
thalamus
effect sizes
1.5
1
0.5
0
-0.5
-1
-1.5
-2
-2.5
-3
-3.5
bias
chosen value
unchosen value
chosen uncertainty
unchosen uncertainty
reversal estimate

S5 **Distinct patterns of change in switching after TUS.** Compared to sham (A), after dmPFC TUS, other than a continuing bias to stick with the same choice trial after trial, animals' switching was only significantly influenced by the probability of reward associated with the choice that they had taken recently (choice value); as this declined monkeys became more likely to switch. Other information such as uncertainty about reward probability estimates (choice uncertainty, alternative uncertainty), the probability of reward for the alternative choice (alternative value), and reversal estimate now had little effect on switching. After TUS of hippocampus (C) switching was relatively more promoted by uncertainty about the recently chosen option's reward probability than by the reversal estimate that the animal might have held (compare relative sizes of third and fifth bars in C as opposed to A). This meant that correlated task performance became similar to performance of the uncorrelated task (Fig.3). (D) Interactions between anterior medial thalamus and dmPFC reflected the integration of reversal estimate and choice value information and after anterior medial thalamic TUS, the normal pattern of integrated impact of reversal estimate and choice value information on switching was reversed. Logistic regression analyses were applied to each session (illustrated by grey dots) from each animal (illustrated in three columns corresponding to each of the three animals) to illustrate the impact of the factors in terms of regression coefficients for each animal and session. Statistical inference was performed in the hierarchical LMEM4.
