# Supplementary material for: Interaction and functional specialization across a distributed neural circuit for flexible task control in macaques
Source: Nat Commun. 2026 Jan 24;17:1063. doi: 10.1038/s41467-025-65766-0 (PMC12852943; doi:10.1038/s41467-025-65766-0)
Supplement: Supplementary file 2 — Reporting Summary [file 41467_2025_65766_MOESM2_ESM.pdf]

Corresponding author(s): Kevin Marche

Last updated by author(s): 7 October 2025

Reporting Summary

Nature Portfolio wishes to improve the reproducibility of the work that we publish. This form provides structure for consistency and transparency in reporting. For further information on Nature Portfolio policies, see our Editorial Policies and the Editorial Policy Checklist.

Statistics

For all statistical analyses, confirm that the following items are present in the figure legend, table legend, main text, or Methods section.

n/a	Confirmed	
☐	☒	The exact sample size (n) for each experimental group/condition, given as a discrete number and unit of measurement
☐	☒	A statement on whether measurements were taken from distinct samples or whether the same sample was measured repeatedly
☐	☒	The statistical test(s) used AND whether they are one- or two-sided<br>*Only common tests should be described solely by name; describe more complex techniques in the Methods section.*
☐	☒	A description of all covariates tested
☐	☒	A description of any assumptions or corrections, such as tests of normality and adjustment for multiple comparisons
☐	☒	A full description of the statistical parameters including central tendency (e.g. means) or other basic estimates (e.g. regression coefficient) AND variation (e.g. standard deviation) or associated estimates of uncertainty (e.g. confidence intervals)
☐	☒	For null hypothesis testing, the test statistic (e.g. F, t, r) with confidence intervals, effect sizes, degrees of freedom and P value noted<br>*Give P values as exact values whenever suitable.*
☐	☒	For Bayesian analysis, information on the choice of priors and Markov chain Monte Carlo settings
☐	☒	For hierarchical and complex designs, identification of the appropriate level for tests and full reporting of outcomes
☐	☒	Estimates of effect sizes (e.g. Cohen's d, Pearson's r), indicating how they were calculated

Our web collection on statistics for biologists contains articles on many of the points above.

Software and code

Policy information about availability of computer code

Data collection	We used Presentation software (Neurobehavioral Systems Inc., Albany, CA) to control the experimental task displayed on the screen and to receive triggers from the MRI scanner. MRI data collection was performed using a 3T horizontal bore MRI scanner coupled with a four-channel phased array receiving coil and a radial transmission coil (Windmiller Kolster Scientific Fresno, CA). We used Brainsight Neuronavigation System (Rogue Research, Montreal, CA) to position the ultrasound transducer on the skull of the animals.
Data analysis	All Bayesian modelling, and behavioural and time course analysis were conducted in MATLAB (MathWorks) using custom analysis scripts. All LMEMs were performed in R. fMRI data processing was performed using a series of custom made scripts using functions from FMRIB Software Library (FSL), Advanced Normalization Tools (http://stnava.github.io/ANTs), the Human Connectome Project Workbench (https://www.humanconnectome.org/software/connectome-workbench), SENSE (Windmiller Kolster Scientific, Fresno, CA), as well as the Magnetic Resonance Comparative Anatomy Toolbox (MrCat; https://github.com/neuroecology/MrCat). The MATLAB custom code supporting the behavioural results of this study is available in the OSF repository https://osf.io/54k9g/.

For manuscripts utilizing custom algorithms or software that are central to the research but not yet described in published literature, software must be made available to editors and reviewers. We strongly encourage code deposition in a community repository (e.g. GitHub). See the Nature Portfolio guidelines for submitting code & software for further information.

Data

Policy information about availability of data

All manuscripts must include a data availability statement. This statement should provide the following information, where applicable:

- Accession codes, unique identifiers, or web links for publicly available datasets
- A description of any restrictions on data availability
- For clinical datasets or third party data, please ensure that the statement adheres to our policy

The processed data and results to reproduce the figures of the paper, have been deposited in the OSF repository https://osf.io/54k9g/.

Research involving human participants, their data, or biological material

Policy information about studies with human participants or human data. See also policy information about sex, gender (identity/presentation), and sexual orientation and race, ethnicity and racism.

Reporting on sex and gender	*Use the terms sex (biological attribute) and gender (shaped by social and cultural circumstances) carefully in order to avoid confusing both terms. Indicate if findings apply to only one sex or gender; describe whether sex and gender were considered in study design; whether sex and/or gender was determined based on self-reporting or assigned and methods used.*<br>*Provide in the source data disaggregated sex and gender data, where this information has been collected, and if consent has been obtained for sharing of individual-level data; provide overall numbers in this Reporting Summary. Please state if this information has not been collected.*<br>*Report sex- and gender-based analyses where performed, justify reasons for lack of sex- and gender-based analysis.*
Reporting on race, ethnicity, or other socially relevant groupings	*Please specify the socially constructed or socially relevant categorization variable(s) used in your manuscript and explain why they were used. Please note that such variables should not be used as proxies for other socially constructed/relevant variables (for example, race or ethnicity should not be used as a proxy for socioeconomic status).*<br>*Provide clear definitions of the relevant terms used, how they were provided (by the participants/respondents, the researchers, or third parties), and the method(s) used to classify people into the different categories (e.g. self-report, census or administrative data, social media data, etc.)*<br>*Please provide details about how you controlled for confounding variables in your analyses.*
Population characteristics	*Describe the covariate-relevant population characteristics of the human research participants (e.g. age, genotypic information, past and current diagnosis and treatment categories). If you filled out the behavioural & social sciences study design questions and have nothing to add here, write "See above."*
Recruitment	*Describe how participants were recruited. Outline any potential self-selection bias or other biases that may be present and how these are likely to impact results.*
Ethics oversight	*Identify the organization(s) that approved the study protocol.*

Note that full information on the approval of the study protocol must also be provided in the manuscript.

Field-specific reporting

Please select the one below that is the best fit for your research. If you are not sure, read the appropriate sections before making your selection.

- [x] Life sciences
- [ ] Behavioural & social sciences
- [ ] Ecological, evolutionary & environmental sciences

For a reference copy of the document with all sections, see nature.com/documents/nr-reporting-summary-flat.pdf

Life sciences study design

All studies must disclose on these points even when the disclosure is negative.

Sample size	Due to the practical and ethical limitations associated with using non-human primates, seven adult male rhesus macaques (Macaca mulatta) were tested.
Data exclusions	fMRI sessions in which the monkey did not complete at least 100 trials within 120 minutes were excluded. Thresholds were pre-established.
Replication	We used stringent statistical tests to ensure the reliability of all results, obtaining consistent outcomes across all subjects. Furthermore, Experiment 2 overlapped with one condition of Experiment 1, and both groups of animals displayed the same behaviour. The ultrasound-related results from Experiment 2 confirmed the fMRI results from Experiment 1.
Randomization	In each experiment, the animals participated in all conditions. In Experiment 2, the order of the stimulation sessions was set in a pseudo-order and counterbalanced across animals. In all experiments, reversals occurred within sessions according to animal performance.
Blinding	There was no group allocation in Experiment 1. In Experiment 2, the animals underwent different stimulation conditions on different days. The experimenters could not be blind to the conditions because they needed to perform TUS on a predetermined location in the brain. However, care was taken to ensure that the subjects' experience was the same in terms of duration, noise and stress.

Behavioural & social sciences study design

All studies must disclose on these points even when the disclosure is negative.

Study description	*Briefly describe the study type including whether data are quantitative, qualitative, or mixed-methods (e.g. qualitative cross-sectional, quantitative experimental, mixed-methods case study).*
Research sample	*State the research sample (e.g. Harvard university undergraduates, villagers in rural India) and provide relevant demographic information (e.g. age, sex) and indicate whether the sample is representative. Provide a rationale for the study sample chosen. For studies involving existing datasets, please describe the dataset and source.*
Sampling strategy	*Describe the sampling procedure (e.g. random, snowball, stratified, convenience). Describe the statistical methods that were used to predetermine sample size OR if no sample-size calculation was performed, describe how sample sizes were chosen and provide a rationale for why these sample sizes are sufficient. For qualitative data, please indicate whether data saturation was considered, and what criteria were used to decide that no further sampling was needed.*
Data collection	*Provide details about the data collection procedure, including the instruments or devices used to record the data (e.g. pen and paper, computer, eye tracker, video or audio equipment) whether anyone was present besides the participant(s) and the researcher, and whether the researcher was blind to experimental condition and/or the study hypothesis during data collection.*
Timing	*Indicate the start and stop dates of data collection. If there is a gap between collection periods, state the dates for each sample cohort.*
Data exclusions	*If no data were excluded from the analyses, state so OR if data were excluded, provide the exact number of exclusions and the rationale behind them, indicating whether exclusion criteria were pre-established.*
Non-participation	*State how many participants dropped out/declined participation and the reason(s) given OR provide response rate OR state that no participants dropped out/declined participation.*
Randomization	*If participants were not allocated into experimental groups, state so OR describe how participants were allocated to groups, and if allocation was not random, describe how covariates were controlled.*

Ecological, evolutionary & environmental sciences study design

All studies must disclose on these points even when the disclosure is negative.

Study description	*Briefly describe the study. For quantitative data include treatment factors and interactions, design structure (e.g. factorial, nested, hierarchical), nature and number of experimental units and replicates.*
Research sample	*Describe the research sample (e.g. a group of tagged Passer domesticus, all Stenocereus thurberi within Organ Pipe Cactus National Monument), and provide a rationale for the sample choice. When relevant, describe the organism taxa, source, sex, age range and any manipulations. State what population the sample is meant to represent when applicable. For studies involving existing datasets, describe the data and its source.*
Sampling strategy	*Note the sampling procedure. Describe the statistical methods that were used to predetermine sample size OR if no sample-size calculation was performed, describe how sample sizes were chosen and provide a rationale for why these sample sizes are sufficient.*
Data collection	*Describe the data collection procedure, including who recorded the data and how.*
Timing and spatial scale	*Indicate the start and stop dates of data collection, noting the frequency and periodicity of sampling and providing a rationale for these choices. If there is a gap between collection periods, state the dates for each sample cohort. Specify the spatial scale from which the data are taken*
Data exclusions	*If no data were excluded from the analyses, state so OR if data were excluded, describe the exclusions and the rationale behind them, indicating whether exclusion criteria were pre-established.*
Reproducibility	*Describe the measures taken to verify the reproducibility of experimental findings. For each experiment, note whether any attempts to repeat the experiment failed OR state that all attempts to repeat the experiment were successful.*
Randomization	*Describe how samples/organisms/participants were allocated into groups. If allocation was not random, describe how covariates were controlled. If this is not relevant to your study, explain why.*
Blinding	*Describe the extent of blinding used during data acquisition and analysis. If blinding was not possible, describe why OR explain why blinding was not relevant to your study.*

Did the study involve field work? ☐ Yes ☐ No

Field work, collection and transport

Field conditions — *Describe the study conditions for field work, providing relevant parameters (e.g. temperature, rainfall).*

Location — *State the location of the sampling or experiment, providing relevant parameters (e.g. latitude and longitude, elevation, water depth).*

Access & import/export — *Describe the efforts you have made to access habitats and to collect and import/export your samples in a responsible manner and in compliance with local, national and international laws, noting any permits that were obtained (give the name of the issuing authority, the date of issue, and any identifying information).*

Disturbance — *Describe any disturbance caused by the study and how it was minimized.*

Reporting for specific materials, systems and methods

We require information from authors about some types of materials, experimental systems and methods used in many studies. Here, indicate whether each material, system or method listed is relevant to your study. If you are not sure if a list item applies to your research, read the appropriate section before selecting a response.

Materials & experimental systems

n/a	Involved in the study	
☐	☐	Antibodies
☐	☐	Eukaryotic cell lines
☐	☐	Palaeontology and archaeology
☐	☒	Animals and other organisms
☐	☐	Clinical data
☐	☐	Dual use research of concern
☐	☐	Plants

Methods

n/a	Involved in the study	
☐	☐	ChIP-seq
☐	☐	Flow cytometry
☐	☒	MRI-based neuroimaging

Antibodies

Antibodies used — *Describe all antibodies used in the study; as applicable, provide supplier name, catalog number, clone name, and lot number.*

Validation — *Describe the validation of each primary antibody for the species and application, noting any validation statements on the manufacturer's website, relevant citations, antibody profiles in online databases, or data provided in the manuscript.*

Eukaryotic cell lines

Policy information about cell lines and Sex and Gender in Research

Cell line source(s) — *State the source of each cell line used and the sex of all primary cell lines and cells derived from human participants or vertebrate models.*

Authentication — *Describe the authentication procedures for each cell line used OR declare that none of the cell lines used were authenticated.*

Mycoplasma contamination — *Confirm that all cell lines tested negative for mycoplasma contamination OR describe the results of the testing for mycoplasma contamination OR declare that the cell lines were not tested for mycoplasma contamination.*

Commonly misidentified lines (See ICLAC register) — *Name any commonly misidentified cell lines used in the study and provide a rationale for their use.*

Palaeontology and Archaeology

Specimen provenance — *Provide provenance information for specimens and describe permits that were obtained for the work (including the name of the issuing authority, the date of issue, and any identifying information). Permits should encompass collection and, where applicable, export.*

Specimen deposition — *Indicate where the specimens have been deposited to permit free access by other researchers.*

Dating methods	*If new dates are provided, describe how they were obtained (e.g. collection, storage, sample pretreatment and measurement), where they were obtained (i.e. lab name), the calibration program and the protocol for quality assurance OR state that no new dates are provided.*

☐ Tick this box to confirm that the raw and calibrated dates are available in the paper or in Supplementary Information.

Ethics oversight	*Identify the organization(s) that approved or provided guidance on the study protocol, OR state that no ethical approval or guidance was required and explain why not.*

Note that full information on the approval of the study protocol must also be provided in the manuscript.

Animals and other research organisms

Policy information about studies involving animals; ARRIVE guidelines recommended for reporting animal research, and Sex and Gender in Research

Laboratory animals	Seven adult male rhesus macaques (Macaca mulatta). They were aged between 9-12 years and weighed between 11-17 kg at the time of the experiments.
Wild animals	the study did not involve wild animals
Reporting on sex	findings apply to only male sex
Field-collected samples	the study did not involve samples collected from the field
Ethics oversight	All procedures were conducted in accordance with the UK Home Office licence issued under the Animal (Scientific Procedures) Act 1986 and the European Union guidelines (EU Directive 2010/63/EU).

Note that full information on the approval of the study protocol must also be provided in the manuscript.

Clinical data

Policy information about clinical studies

All manuscripts should comply with the ICMJE guidelines for publication of clinical research and a completed CONSORT checklist must be included with all submissions.

Clinical trial registration	*Provide the trial registration number from ClinicalTrials.gov or an equivalent agency.*
Study protocol	*Note where the full trial protocol can be accessed OR if not available, explain why.*
Data collection	*Describe the settings and locales of data collection, noting the time periods of recruitment and data collection.*
Outcomes	*Describe how you pre-defined primary and secondary outcome measures and how you assessed these measures.*

Dual use research of concern

Policy information about dual use research of concern

Hazards

Could the accidental, deliberate or reckless misuse of agents or technologies generated in the work, or the application of information presented in the manuscript, pose a threat to:

No	Yes	
☐	☐	Public health
☐	☐	National security
☐	☐	Crops and/or livestock
☐	☐	Ecosystems
☐	☐	Any other significant area

Experiments of concern

Does the work involve any of these experiments of concern:

No	Yes	
☐	☐	Demonstrate how to render a vaccine ineffective
☐	☐	Confer resistance to therapeutically useful antibiotics or antiviral agents
☐	☐	Enhance the virulence of a pathogen or render a nonpathogen virulent
☐	☐	Increase transmissibility of a pathogen
☐	☐	Alter the host range of a pathogen
☐	☐	Enable evasion of diagnostic/detection modalities
☐	☐	Enable the weaponization of a biological agent or toxin
☐	☐	Any other potentially harmful combination of experiments and agents

Plants

Seed stocks	*Report on the source of all seed stocks or other plant material used. If applicable, state the seed stock centre and catalogue number. If plant specimens were collected from the field, describe the collection location, date and sampling procedures.*
Novel plant genotypes	*Describe the methods by which all novel plant genotypes were produced. This includes those generated by transgenic approaches, gene editing, chemical/radiation-based mutagenesis and hybridization. For transgenic lines, describe the transformation method, the number of independent lines analyzed and the generation upon which experiments were performed. For gene-edited lines, describe the editor used, the endogenous sequence targeted for editing, the targeting guide RNA sequence (if applicable) and how the editor was applied.*
Authentication	*Describe any authentication procedures for each seed stock used or novel genotype generated. Describe any experiments used to assess the effect of a mutation and, where applicable, how potential secondary effects (e.g. second site T-DNA insertions, mosiacism, off-target gene editing) were examined.*

ChIP-seq

Data deposition

☐ Confirm that both raw and final processed data have been deposited in a public database such as GEO.

☐ Confirm that you have deposited or provided access to graph files (e.g. BED files) for the called peaks.

Data access links<br>*May remain private before publication.*	*For "Initial submission" or "Revised version" documents, provide reviewer access links. For your "Final submission" document, provide a link to the deposited data.*
Files in database submission	*Provide a list of all files available in the database submission.*
Genome browser session<br>(e.g. UCSC)	*Provide a link to an anonymized genome browser session for "Initial submission" and "Revised version" documents only, to enable peer review. Write "no longer applicable" for "Final submission" documents.*

Methodology

Replicates	*Describe the experimental replicates, specifying number, type and replicate agreement.*
Sequencing depth	*Describe the sequencing depth for each experiment, providing the total number of reads, uniquely mapped reads, length of reads and whether they were paired- or single-end.*
Antibodies	*Describe the antibodies used for the ChIP-seq experiments; as applicable, provide supplier name, catalog number, clone name, and lot number.*
Peak calling parameters	*Specify the command line program and parameters used for read mapping and peak calling, including the ChIP, control and index files used.*
Data quality	*Describe the methods used to ensure data quality in full detail, including how many peaks are at FDR 5% and above 5-fold enrichment.*
Software	*Describe the software used to collect and analyze the ChIP-seq data. For custom code that has been deposited into a community repository, provide accession details.*

Flow Cytometry

Plots

Confirm that:

- [ ] The axis labels state the marker and fluorochrome used (e.g. CD4-FITC).
- [ ] The axis scales are clearly visible. Include numbers along axes only for bottom left plot of group (a 'group' is an analysis of identical markers).
- [ ] All plots are contour plots with outliers or pseudocolor plots.
- [ ] A numerical value for number of cells or percentage (with statistics) is provided.

Methodology

Sample preparation	*Describe the sample preparation, detailing the biological source of the cells and any tissue processing steps used.*
Instrument	*Identify the instrument used for data collection, specifying make and model number.*
Software	*Describe the software used to collect and analyze the flow cytometry data. For custom code that has been deposited into a community repository, provide accession details.*
Cell population abundance	*Describe the abundance of the relevant cell populations within post-sort fractions, providing details on the purity of the samples and how it was determined.*
Gating strategy	*Describe the gating strategy used for all relevant experiments, specifying the preliminary FSC/SSC gates of the starting cell population, indicating where boundaries between "positive" and "negative" staining cell populations are defined.*

- [ ] Tick this box to confirm that a figure exemplifying the gating strategy is provided in the Supplementary Information.

Magnetic resonance imaging

Experimental design

Design type	task based, event related
Design specifications	Experiment 1 consisted of 16–18 sessions per subject. These sessions were divided into two conditions according to the correlation of the reward schedules for each option. For each trial, stimuli were presented for up to 10 seconds until a response was made. The mean duration of the action-outcome delay was 3.394 seconds (variance 0.605 seconds; uniform distribution). The outcome cue lasted two seconds. The inter-trial interval lasted between 6.5 and 7.5 seconds (uniform distribution).
Behavioral performance measures	The choices and reaction times were measured. These were modulated by the compound values of the two alternative options and by each of their component features. This showed that the animals were performing the task correctly.

Acquisition

Imaging type(s)	Functional, structural
Field strength	3 Tesla
Sequence & imaging parameters	Structural images were acquired under general anaesthesia prior to the experiment, using a T1-weighted MP-RAGE sequence with a solution of 0.5 x 0.5 x 0.5 mm, repetition time (TR) = 2.5 seconds, echo time (TE) = 4.04 ms, inversion pulse time (TI) = 1.1s, and flip angle of 8°.<br>fMRI data were collected during task performance with a gradient-echo T2* echo planar imaging (EPI) sequence with a resolution of 1.5 x 1.5 x 1.5 mm, interleaved slice acquisition, TR = 2.28s, TE = 30ms, and flip angle of 90°. Acceleration facto 2.<br>At the end of each session, to aid image reconstruction, a proton-density-weighted image was acquired using a gradient-refocused-echo (GRE) sequence with a resolution of 1.5 x 1.5 x 1/5 mm, TR = 10ms, TE = 2.52ms, and flip angle 25°.
Area of acquisition	whole brain

Diffusion MRI [ ] Used [x] Not used

Preprocessing

Preprocessing software	fMRI images were pre-processed using SENSE (Windmiller Kolster Scientific), Advanced Normalisation Tools (ANTs) version 2.2.0, Magnetic Resonance Comparative Anatomy Toolbox (MrCat – https://github.com/neuroecology/MrCat) and tools of

	FMRIB Software Library (FSL) version 6.0.0, namely BET, FAST, FLIRT, and FNIRT. Code written in Bash.
Normalization	The slice-registered average functional image was non-linearly registered to the high-resolution structural reference of each subject, and then this was registered to the group-specific template using tools from ANTs as implemented in MrCat.
Normalization template	A T1w group template specific to the set of subjects was constructed using two iterations of (i) registration to an initial template in F99 space (Van Essen, 2002; Van Essen and Dierker, 2007), (ii) group averaging, (iii) registration to the new group template. This was accomplished using tools from ANTs as implemented in MrCat.
Noise and artifact removal	To correct for non-linear motion-related artefact in the phase-encoding direction due to body motion, each slice was registered, first linearly, then non-linearly, to a robust reference based on EPI volumes from the same timeseries with least distortion, using a processing pipeline implemented in MrCat. To avoid overlifting, the degrees of freedom were constrained in several ways: only distortions along the phase-encoding direction were considered; registration was initialised using priors from temporally neighbouring slices; low-order solutions were preferred over high-order registration (rigid>affine>non-linear); non-linear degrees of freedom were regularised using b-splines.<br>The functional images were temporally filtered with high-pass temporal filtering, 3-dB cutoff of 100s, and spatially smoothed with Gaussian spatial smoothing, full-width half maximum of 3mm.
Volume censoring	During analysis (in FEAT by FSL), low-quality EPI volumes, suffering from strong artefacts, were excluded. Volume quality was assessed based on (i) slice-registration cost (the normalised correlation between the current volume and the robust average after optimal registration), (ii) linear scaling along the phase-encoding direction (directly related to signal intensity loss due to motion distortion), (iii) non-linear deformation (penalising volumes that require highly non-linear deformations).

Statistical modeling & inference

Model type and settings	We employed a univariate approach within the general linear model framework to perform whole-brain statistical analyses of functional data as implemented in the FMRIB Software Library. Using this framework we initially performed a first-level fixed effects analysis to process each individual experimental session which were then combined in a second-level mixed-effects analysis (FLAME 1 + 2) treating sessions as a random effect.
Effect(s) tested	In Experiment 1:<br>- Parametric regressor representing the difference between chosen stimulus value and unchosen stimulus value.<br>- Parametric regressor representing the difference between chosen stimulus uncertainty and unchosen stimulus uncertainty.<br>- Parametric regressor representing the uncertainty weighted probability of reversal from the current latent task state to the other.<br>The full models are described in the Methods.

Specify type of analysis: ☐ Whole brain ☐ ROI-based ☒ Both

Statistic type for inference<br>(See Eklund et al. 2016)	Clusters were determined using a voxel threshold of Z>2.3
Correction	FEW-corrected threshold of P=0.05

Models & analysis

n/a	Involved in the study
☒	☐ Functional and/or effective connectivity
☒	☐ Graph analysis
☒	☐ Multivariate modeling or predictive analysis

Functional and/or effective connectivity	*Report the measures of dependence used and the model details (e.g. Pearson correlation, partial correlation, mutual information).*
Graph analysis	*Report the dependent variable and connectivity measure, specifying weighted graph or binarized graph, subject- or group-level, and the global and/or node summaries used (e.g. clustering coefficient, efficiency, etc.).*
Multivariate modeling and predictive analysis	*Specify independent variables, features extraction and dimension reduction, model, training and evaluation metrics.*
